# “Recurrent Fibroid” Cured by Silicea in a High Potency

**Published:** 1884-07

**Authors:** J. T. Kent


### "RECURRENT FIBROID" CURED BY SILICEA IN HIGH POTENCY.

Frank H., a compositor in the *Globe-Democrat* office, St. Louis, came to my office to have a tumor removed by the knife. It had been removed twice and was called a *recurrent fibroid*. It was the size of a hen's egg and very hard, located in the left side of the neck, not connected with the parotid, through growing a little below it. I advised him to give me time to prepare him for removal. I took his symptoms and found that he was better by wrapping up even the head. He was timid in going into a new enterprise, though abundantly able to perform the task. *He lacked confidence in his own ability, yet when he had begun he would do well.*

He took Silicea 5m., April 1st, 1883. Six weeks later he called, and the tumor was reduced one half. Sil. 72m., dry, one dose. Six weeks later almost gone. January 23d, 1884, Sil. 72m., one dose. The tumor has disappeared. This prescribing has been commented upon by a large number of friends, who think the one dose business a mystery. He got no Sac. Lac., as I had his confidence. I did not prescribe for the tumor, but for the patient. My prescription could not have been different had the tumor not been present.

The tumor was not included in the totality of symptoms, as it was not a symptom; it furnished no part of the guide to a remedy. The symptoms expressive of the whole state existed prior to the tumor, and it was the language of this pre-existing state that I read, as out of this pre-existing state, grew the tumor. I must interpret the language or expressions of *cause*, not *effect*. The man who is guided by pathology can use the knife. To use the knife is but to acknowledge one's ignorance of a method by which he can avoid cutting.—*J. T. Kent, in Medical Advance.*

